# LASSO type penalized spline regression for binary data

**DOI:** 10.1186/s12874-021-01234-9

**Published:** 2021-04-24

**Authors:** Muhammad Abu Shadeque Mullah, James A. Hanley, Andrea Benedetti

**Affiliations:** 1 Department of Epidemiology, Biostatistics and Occupational Health, McGill University, Montreal, Canada; 2 Department of Medicine, McGill University, and Respiratory Epidemiology & Clinical Research Unit, Montreal Chest Institute, McGill University Health Centre, Montreal, Canada

**Keywords:** Penalized splines, Generalized linear mixed models, Ridge regression, Least absolute shrinkage and selection operator (LASSO), Markov chain Monte Carlo

## Abstract

**Background:**

Generalized linear mixed models (GLMMs), typically used for analyzing correlated data, can also be used for smoothing by considering the knot coefficients from a regression spline as random effects. The resulting models are called semiparametric mixed models (SPMMs). Allowing the random knot coefficients to follow a normal distribution with mean zero and a constant variance is equivalent to using a penalized spline with a ridge regression type penalty. We introduce the least absolute shrinkage and selection operator (LASSO) type penalty in the SPMM setting by considering the coefficients at the knots to follow a Laplace double exponential distribution with mean zero.

**Methods:**

We adopt a Bayesian approach and use the Markov Chain Monte Carlo (MCMC) algorithm for model fitting. Through simulations, we compare the performance of curve fitting in a SPMM using a LASSO type penalty to that of using ridge penalty for binary data. We apply the proposed method to obtain smooth curves from data on the relationship between the amount of pack years of smoking and the risk of developing chronic obstructive pulmonary disease (COPD).

**Results:**

The LASSO penalty performs as well as ridge penalty for simple shapes of association and outperforms the ridge penalty when the shape of association is complex or linear.

**Conclusion:**

We demonstrated that LASSO penalty captured complex dose-response association better than the Ridge penalty in a SPMM.

**Supplementary Information:**

The online version contains supplementary material available at (10.1186/s12874-021-01234-9).

## Background

The association between the level of a continuous variable and the mean response at that level may take any functional form. To reduce bias resulting from mis-specifying the functional form and also from the loss of efficiency in testing induced by categorizing continuous variables, the use of nonparametric (flexible) regression models is often recommended to model the effect of variables recorded on a continuous scale [1, 2].

Nonparametric regression techniques, by their nature, do not require any parametric representation to be specified a priori, and instead determine the shape of the association directly from the data. While several competing approaches are available for such modeling (see, e.g., [3–14]), we focus on penalized splines (P-splines) which is a powerful technique to fit a smooth curve to the data in a scatterplot. In P-splines, a greater degree of smoothness is achieved by specifying a large number of knots and imposing restrictions on the knot coefficients to prevent over-fitting [15]. An important issue, however, is to select a suitable value for the smoothing parameter, which is not a trivial task.

Penalized splines can be viewed as a particular case of generalized linear mixed models (GLMMs). To achieve a smooth function, the GLMM can be used to shrink the regression coefficients of knot points from a regression spline towards zero, by including them as random effects and constraining them to follow a normal distribution with mean zero and constant variance. The resulting models are called semiparametric mixed models (SPMMs) [13]. The main advantage of this approach is that the smoothing parameter, which controls the trade-off between bias and variance, may be directly estimated from the data [16]. Moreover, we can take full advantage of existing methods and software for GLMMs.

Restricting the changes in the slope at the knots to follow a normal distribution with mean zero and constant variance is equivalent to using a penalized spline by imposing a ridge penalty (*L*_2_ penalization), that is, restricting the sum of squares of the spline coefficients at knot points to be less than a judiciously selected constant [16]. However, imposing *L*_1_ penalization (i.e., restricting the sum of absolute values of the knot coefficients) is also possible. By constraining the coefficients at knots to follow independent and identical Laplace (i.e., double exponential) distributions with means zero will give rise to a LASSO type penalty in a SPMM setting.

A LASSO type penalty has previously been used in a penalized spline setting (see, for example, [17–22]) but primarily for variable selection. The literature on curve fitting by LASSO penalty is sparse; to our knowledge, it has never been used or investigated in a SPMM setting for non-Gaussian outcomes. Because of the nature of the LASSO constraint, it shrinks some coefficients and sets others to zero, and hence may be hypothesized to produce a smoother fit.

In this paper we introduce the LASSO type penalty under the SPMM framework of curve fitting and investigate if the performance of curve fitting by SPMM can be improved using the LASSO penalty rather than using a typical ridge penalty. For estimation we adopt the Bayesian approach and use Markov Chain Monte Carlo (MCMC) algorithm. Recent development in Bayesian computational software has facilitated smoothing under full Bayesian framework via mixed model representation of penalized splines (see, [13, 15, 16, 23–28]).

We consider binary responses and smoothing of a single continuous covariate, and systematically compare the performance of curve fitting using two penalties (LASSO and ridge), by simulation. We apply the proposed method to estimate the effect of (amount of cigarette) smoking on the risk of developing COPD.

## Methods

### Penalized spline models for binary data

We introduce the idea of penalized spline regression with the following simple logistic model:

1 $$\begin{array}{@{}rcl@{}} \text{logit}\ \mathbb{P}\left[Y_{i} = 1\mid x_{i}\right] = m(x_{i}), \quad i = 1, \dots, n, \end{array} $$

where *Y*_*i*_ is a binary response variable, *x*_*i*_ a continuous covariate measured on subject *i* and *m*(·) is a smooth function. To estimate *m*(*x*) we use low-rank thin-plate (LRTP) splines [29] with *K* knots $t_{1}, \dots, t_{K}$ as given by

2 $$\begin{array}{@{}rcl@{}} m(x)=\beta_{0}+\beta_{1} x+ \sum\limits_{k=1}^{K}u_{k} b_{k}(x), \end{array} $$

where $\beta _{0}, \beta _{1}, u_{1}, \dots, u_{K}$ are regression coefficients. For $k = 1, \dots, K$, the basis functions are:

$$\begin{array}{@{}rcl@{}} b_{k}(x) &=& \sum\limits_{i=1}^{K}|x-t_{i}|^{3} w_{i}(k) \end{array} $$

in which *w*_*i*_(*k*) is the (i, k)th element of the penalty matrix $\Omega _{K}^{-1/2}$, where the (i, k)th entry of *Ω*_*K*_ is |*t*_*i*_−*t*_*k*_|^3^ for 1≤*i,k*≤*K*. The LRTP has the advantage of using a relatively small number of knots to obtain a smoother fit. It also has good mixing properties in the MCMC analysis (see, e.g., [15]). Other basis functions that are often used include truncated polynomial splines [16], natural cubic splines[30], B-splines [12], and thin plate regression splines [29].

Denoting $ {Y} = (Y_{1}, \dots, Y_{n})^{T}, {X} = \left [1, x_{i}\right ]_{1\le i \le n}, {Z} = \left [b_{1}(x_{i}), \dots, b_{K}(x_{i})\right ]_{1\le i \le n}, {\beta }= (\beta _{0}, \beta _{1})^{T}$ and ${u}= (u_{1}, \dots, u_{K})^{T}$, Eqs. 1 and (2) can be written more compactly in matrix notation as

3 $$\begin{array}{@{}rcl@{}} \text{logit}\ \mathbb{P}\left[{Y} = 1\mid {X}, {Z}\right] = {X} {\beta} + {Z} {u}. \end{array} $$

Model (3) is purely parametric and easily estimated as an ordinary logistic regression model. This approach is known as regression spline smoothing. Here, the *u*_*k*_ represent changes in slope from one segment to the next. So, unconstrained estimation of the *u*_*k*_ would lead to a “overly fluctuating" fit due to the large number of truncated polynomials. An optimum fit could be achieved by imposing a penalty on the spline coefficients. Specifically, one could choose a large number of knots (typically 5 to 20, as suggested by Ruppert [31]) and prevent over-fitting by putting a constraint on the spline coefficients. Constraints that can be imposed on *u*_*k*_ are: (i) $\sum u_{k}^{2} \le c$, (ii) $\sum \lvert u_{k} \rvert \le c$, and (iii) max |*u*_*k*_|≤*c*. Here, *c*≥0 is the tuning parameter. Restrictions (i) and (ii) are known as ‘ridge’ and ‘LASSO’ type penalties, respectively [31, 32]. Each of these constraints will lead to a smoother fit for an appropriate choice of *c*. However, the ridge penalty is used most frequently as it is much easier to implement.

Denoting ${\theta } = \left [\mathbb {\beta }, u \right ]^{T} $ and *W*=[*X,Z*]=[*w*_*i*_]_1≤*i*≤*n*_, we can write (for $i = 1, \dots, n$)

4 $$\begin{array}{@{}rcl@{}} \pi_{i} = \mathbb{P}\left[Y_{i} = 1\mid w_{i}\right] = \frac{1}{1 + \exp(-w_{i}^{T}{\theta})}. \end{array} $$

The log likelihood function for *θ* is given by

5 $$\begin{array}{@{}rcl@{}} l({\theta}) = - \sum\limits_{i=1}^{n}\left[(1-y_{i})w_{i}^{T}{\theta} + \ln \left(1+ \exp(-w_{i}^{T}{\theta}) \right) \right]. \end{array} $$

The LASSO constraint $\sum \lvert u_{k} \rvert \le c$ is equivalent to the addition of a penalty term $\lambda \sum \limits _{k=1}^{K}\lvert {u_{k}} \rvert $ to the joint log-likelihood of *θ* so that the constrained log-likelihood function is

6 $$ \begin{aligned} l_{CL}({\theta}) = - \sum\limits_{i=1}^{n}\left[(1-y_{i})w_{i}^{T}{\theta} + \ln \left(1+ \exp(-w_{i}^{T}{\theta}) \right) \right] - \lambda \sum\limits_{k=1}^{K}\lvert {u_{k}} \rvert, \end{aligned}  $$

where *λ*≥0 is the smoothing parameter which controls the trade-off between the goodness of fit and smoothness of the estimated curves. The *λ* can be either selected by the user or chosen via numerous methods including cross-validation, generalized cross-validation and a variant of Stein’s unbiased estimate of risk [32].

Similarly, imposing the typical ridge penalty $\sum u_{k}^{2} \le c$ yields a restricted maximization equation as

7 $$ \begin{aligned} l_{CR}({\theta}) = - \sum\limits_{i=1}^{n}\left[(1-y_{i})w_{i}^{T}{\theta} + \ln \left(1+ \exp(-w_{i}^{T}{\theta}) \right) \right] - \lambda \sum\limits_{k=1}^{K} u_{k}^{2}. \end{aligned}  $$

### Bayesian approach to penalized spline

Tibshirani [32] noted that |*u*_*k*_| in (6) is proportional to the negative log-density of a Laplace (double-exponential) distribution. Therefore, the LASSO penalized spline estimate can be obtained as the Bayes posterior mode under independent double-exponential, *DE*(0,*τ*) priors for the *u*_*k*_ with mean 0 and variance 2*τ*^2^,

8 $$\begin{array}{@{}rcl@{}} f(u_{k}\mid \tau) = \frac{1}{2 \tau} \exp\left(-\frac{\lvert {u_{k}}\rvert}{\tau} \right) \end{array} $$

with *λ*=1/*τ*. Again, ${u^{2}_{k}}$ in (7) is proportional to the negative log-density of a normal distribution. As a result, the Bayesian analogue of the ridge regression type penalization involves using normal priors for the *u*_*k*_’s,

9 $$\begin{array}{@{}rcl@{}} p(u_{k} \mid \sigma^{2}) = \frac{1}{\sqrt{2\pi \sigma^{2}}} \exp \left(- \frac{u_{k}^{2}}{2 \sigma^{2}}\right), \quad k = 1, \dots, K. \end{array} $$

with *λ*=1/2*σ*^2^. A fully Bayesian hierarchical modeling involves specifying a hyper prior distribution for *λ*. Typically, a non-informative prior that guarantees a unimodal full posterior is recommended (see, e.g., [33]).

In general, a Bayesian approach for penalized spline involves a prior distribution on *u*_*k*_ specifying that each *u*_*k*_ is likely to be near 0 which is encoded by the mean of 0 for the prior distribution. By shrinking *u*_*k*_ towards zero, the changes in gradient between consecutive line (or curve) segments are reduced to achieve a greater degree of smoothness.

The use of a double-exponential prior on the knot coefficients puts more mass near 0 and in the tails as compared to that of using a Gaussian prior. This reflects the greater tendency of the LASSO penalty to produce estimates that are either 0 or large. As a result, the LASSO penalty shrinks those knot coefficients with minimal values towards 0 (generally faster than the ridge penalty).

#### Penalized splines as mixed models

Using any of the priors (8) and (9), the logistic regression spline model (3) is in fact a logistic mixed effects model

10 $$\begin{array}{@{}rcl@{}} \text{logit}\ \mathbb{P}\left[{Y} =1\mid {X}, {Z}\right] &=& {X} {\beta} + {Z} {u}, \\  {u} &\sim& f({0}, \Lambda_{\gamma}) \end{array} $$

for response vector *Y*, known design matrices *X* and *Z*, fixed effects parameter vector *β*, random effects vector *u*, and a diagonal variance covariance matrix *Λ*_*γ*_=*γ*^2^*I*_*K*_ in which *γ*^2^ is the variance of *u*_*k*_. Thus, the nonlinear association between an outcome and covariates can be modeled using penalized splines within the framework of a mixed effects model, which allows us using current methodology and software for GLMMs. The main advantage of this approach is that the smoothing parameter can be estimated directly from the data in a maximum likelihood or Bayesian framework. Moreover, using a single model, we can analyze correlated and over-dispersed data by adding random effects to the additive predictor, while estimating nonlinear covariate effects by penalized splines. Note that this penalized splines approach can be easily extended to any outcome distribution that belongs to an exponential family.

The likelihood estimation of GLMMs involves a high dimensional integral over the unobserved random effects. In general, the likelihood does not have a closed-form as the integral is intractable, and has to be approximated or evaluated numerically. Two popular approximation techniques are penalized quasi-likelihood (PQL) [34] and (full) Laplace approximation [35]. However, both methods yield biased estimates in curve fitting under SPMM framework, especially for binary data [36, 37]. More refined approximation methods using adaptive Gaussian quadrature are not feasible as the GLMMs representation of the penalized splines involve a large number of random effects [38]. An attractive alternative to likelihood-based approximations is to pursue a Bayesian approach that enjoys exact inference under Bayesian machinery. Bayesian methods have good frequentist properties when the model is correct but are known to be computationally intensive. Moreover, they require specification of prior distributions which is often not a trivial task, especially for variance components (see, e.g., [39]).

Nevertheless, Crainiceanu et al. [15] strongly recommend Bayesian methods for penalized splines by noting at least two potential problems of using approximated likelihood-based estimation. First, the approximation can have a considerable effect on parameter estimation, especially on the variance components. Secondly, the confidence intervals are obtained by replacing the estimated parameters instead of the true parameters and ignoring the inherent additional variability. This results in narrower (than they should be) confidence intervals. We therefore adopt a Bayesian approach to fit the SPMMs in this paper.

#### Bayesian estimation

Bayesian analysis considers all unknown parameters as random variables and characterizes any previous knowledge about parameters by assigning prior distributions to them preceding the data collection. The marginal posterior distribution of parameters given the data are then used as the basis of inference. The posterior densities are, however, analytically unavailable in many cases, especially for complex models. In such cases, the Markov Chain Monte Carlo (MCMC) procedure is used to make inferences by drawing samples from all posterior distributions of interest and calculating the posterior means, medians, quantile-based confidence bands and predictive distributions.

*Prior Specification for Fixed Effects* For each element of the fixed effects vector *β*, we consider $\beta _{j} \thicksim N(0, \sigma ^{2}_{j})$, where $\sigma ^{2}_{j}$ is a large constant to regard the prior as noninformative. We take $\sigma ^{2}_{j} = 10^{6}$ to ensure a proper joint posterior distribution of the parameters under appropriate priors for the variance components.

*Priors for Variance Components* The variance component estimates in Bayesian mixed models are sensitive to the prior specification [39]. For the SPMMs, it is therefore crucial to choose appropriate priors for variance components as curve estimation greatly depends on the variance components. For the variance components *γ*∈{*τ*,*σ*} in (10), perhaps the most popular choice is a highly dispersed inverse-gamma (IG) prior. However, for estimating SPMMs, the IG prior under-estimates the variance parameters and over-smooth the nonparametric functions [36, 37]. Gelman [39] suggested using a wide ranged uniform prior density on variance parameters *γ*, for example,

11 $$\begin{array}{@{}rcl@{}} \gamma \thicksim \mathrm{U}(0, 100). \end{array} $$

In case *γ* is very close to zero, Gelman [39] suggested using a special case of the half-t distribution with df = 1, known as the half-Cauchy distribution:

12 $$\begin{array}{@{}rcl@{}} P(\gamma) \propto (\gamma^{2} + s^{2})^{-1} \end{array} $$

with a large value for the scale parameter *s*, for example, *s*=25. Later on, in the simulation scenarios, we try out several of these different priors and investigate the influence of those.

*Markov Chain Monte Carlo Inference* Assuming independence of prior distributions, the joint posterior distribution of *θ*=(*β*,*u*) and *γ* is given by

13 $$\begin{array}{@{}rcl@{}} P(\theta, \gamma \mid y) &\propto& P(\theta \mid \gamma) P(\gamma) \prod\limits_{i=1}^{n}p(y_{i}\mid \theta, \gamma)\\  &\propto& P(\beta) P(u \mid \gamma) P(\gamma) \prod\limits_{i=1}^{n}p(y_{i}\mid \theta, \gamma).\\  \end{array} $$

Our main interests are to find the posterior marginal distributions *p*(*θ*|*y*) and *p*(*γ*|*y*). The joint posterior (13) does not have a closed form in most cases, and even if it does, we have to perform multiple integration to obtain the marginal distribution for each coefficient of the parameter vectors *θ* and *γ*. These integrals are analytically intractable for most problems. Moreover, the large dimensionality of the integrals hinders the use of numerical integration. A standard solution is to apply MCMC to draw samples from (13) to approximate (the properties of) the marginal posterior distributions of each parameter. A thorough coverage of the MCMC algorithm is provided by Gilks, Richardson and Spiegelhalter [40].

While several software platforms (such as WinBUGS, OpenBUGS, JAGS, INLA, STAN) are now available for GLMM fitting via MCMC sampling, we use JAGS (Just Another Gibbs Sampler) [41] to fit Bayesian models. JAGS is a mature and declarative language for Bayesian model fitting with reasonable computation time and a nice link to R. We call JAGS from inside of R using the R package R2jags [42] and export results to R. Other alternatives to R2jags include rjags [43] and runjags [44].

#### Frequentist methods for curve fitting

We also consider two popular frequentist approaches: (i) Generalized Additive Model (GAM) of Hastie and Tibshirani [10] as implemented in R package gam [45] (ii) GAM of Wood [11] as implemented in R package mgcv [46].

The gam package uses a back-fitting algorithm for model fitting in which the non-parametric smoothing terms are represented by local regression or smoothing splines. The amount of smoothing is controlled by the user-specified degrees of freedom (df). In gam, few df results in a less bumpy but possibly more biased estimate, while many df results in a more flexible curve, with increased risk of over-fitting [10]. The default df in gam is the trace of the implicit smoother matrix minus 1.

In mgcv package, the model is estimated by maximizing a quadratically penalized likelihood. The smooth functions are represented by penalized regression splines using optimal basis functions. The basis dimension (or number of knots) are user-specified and are chosen to be neither too small to avoid over-smoothing nor too large to avoid computational cost. The default basis dimension in mgcv is arbitrary. The smoothing parameter is selected by Generalized Cross Validation (GCV) [47] or Un-Biased Risk Estimator (UBRE)[14] or Akaike Information Criteria (AIC) [48] or Laplace approximation to Restricted Maximum Likelihood (REML) [14] or by regression splines with fixed degrees of freedom, although the REML appeared to be most effective choice [46]. The confidence interval is obtained by employing a Bayesian approach to variance estimation.

## Evaluation of performance

To systematically compare the performance of smoothing in a SPMM using LASSO type penalty versus ridge regression penalty (SPMM-LASSO vs. SPMM-RIDGE), we carried out a series of simulations with data simulated from and analyzed using logistic regression models with smooth terms.

### Methods

#### Data generation

Data were generated from a binary distribution considering three different shapes of association between the probability of a positive outcome and covariate. We considered the sample size of *n*=500. For each configuration, 1,000 datasets were simulated.

Using one independent continuous covariate *x*, binary responses (Ys) were generated according to the model

14 $$\begin{array}{@{}rcl@{}} \text{logit} \mathbb{P}\left[Y_{i} = 1\mid x_{i}\right] = m(x_{i}), \end{array} $$

where the covariate *x* was simulated from a *U*(0,1) and the smooth function *m*(·) took one of the three test functions shown in Table 1. The functions were scaled so that the success probability was in the range [0.02,0.98]. The overall prevalence of a positive (*Y*=1) outcome was kept at 0.5. We considered one simple curve (concave), one complex curve (double hump), and one linear function as the functional form for the association between the covariate and the probability of a positive outcome. A linear function was chosen to verify how well the smooth function recaptured it as a check similar to whether the nominal level of significance (probability of type I error) holds in the hypothesis testing.

**Table 1:** Test functions used for data generation

Name	Shape	Function
Linear	[JWFFXGRAPHICS]s12874-021-01234-9tmc1.eps[JWFFXGRAPHICS]	log(3)∗*x*
Concave	[JWFFXGRAPHICS]s12874-021-01234-9tmc2.eps[JWFFXGRAPHICS]	*sin*(*π*∗*x*)
Double Hump	[JWFFXGRAPHICS]s12874-021-01234-9tmc3.eps[JWFFXGRAPHICS]	$\frac {1}{10} \left \{\frac {6 x^{29}(1-x)^{16}}{\beta (30, 17)} + \frac {4 x^{2}(1-x)^{10}}{\beta (3, 11)} \right \}$

#### Analysis of simulated datasets

Each simulated dataset was analysed by fitting a logistic (mixed effects) model of the form (14) in which the smooth term was represented by a penalized spline using LASSO or ridge penalty. We adopted low-rank thin-plate splines with several knot points. For penalized splines, the number of parameters to be penalized are represented by the number of knots, and the magnitude and number of parameters to be penalized have important consequences. Thus, we considered three different number of knot points: 7, 20 and 35. These choices of knots were following Harrel [30], Rupert [31] and Wand [49]. Harrell [30] argued that using 4-7 knots usually results in a reasonable fit if the knots partition the data into evenly sized groups, whereas Ruppert [31] recommended taking a large number of knots (typically 5 to 20) to ensure the desired flexibility. Wand [49] suggested choosing the number of knots (*K*) as

$$K = \text{min}(\text{number of unique x's}/4, 35)$$

and specifying the knot positions as

15 $$ \begin{aligned} t_{k} = \left(\frac{k+1}{K+2}\right) \text{th sample quantile of unqiue x's}, \qquad 1 \le k \le K. \end{aligned}  $$

Representing the penalized spline as mixed model component, we estimated the model parameters using a Bayesian approach where noninformative priors were used for all parameters. Specifically, *N*(0,10^6^) distributions were used for all fixed effects, while a Uniform (0, 100) prior specification was considered for each variance component. We also fitted models using Half-Cauchy prior with scale parameter set to 25 (i.e., Half-Cauchy (25)) for each variance component and check the sensitivity of the results to this choice. The Bayesian estimates were medians from 55,000 iterations of the MCMC algorithm after discarding the first 5,000 iterations as burn-in. We ran a single chain and thinned it by keeping every 50th iteration. All simulations and analyses were carried out in R software and the MCMC was performed using JAGS (see, Supplementary Material for R code).

For each of the simulated dataset, we also adopted two frequentist GAMs as implemented in R packages: (i) gam, and (ii) mgcv. In gam, we considered smoothing splines for estimating smooth functions and used four different df: 7, 20, 35 and using default. In the mgcv package, the smooth functions were represented using penalized thin plate regression splines. The smoothing parameter was estimated during model fitting by REML. We specified four different number of basis dimensions: 7, 20, 35 and using default.

#### Measures of performance

The overall performance of the estimator $\hat {m}(x)$ was evaluated using the following criteria: (i) mean average squared distance/error (MASE) from the true curves; (ii) pointwise 95% mean average coverage probabilities (MACPs); and (iii) pointwise 95% mean average confidence interval lengths (MACLs).

The pointwise MASE was defined as the mean over the 1,000 replicated datasets of the average squared error,

$$\begin{array}{@{}rcl@{}} \text{ASE} = \left(1/n \right) \sum\limits_{i=1}^{n} \{ \hat{m}(x_{i})-m(x_{i}) \}^{2}. \end{array} $$

The 95% pointwise MACP and MACL were obtained as the means of the 1,000 average coverage probabilities (ACP) and average credible intervals lengths (ACL), respectively. We defined

$$\begin{array}{@{}rcl@{}} \text{ACP} &=& \left(1/n\right) \sum\limits_{i=1}^{n} \mathbf{1} \left(\hat{m}_{L}(x_{i}) < m(x_{i}) < \hat{m}_{U}(x_{i})\right),\\ \text{ACL} &=& \left(1/n\right) \sum\limits_{i=1}^{n} \left(\hat{m}_{U}(x_{i})- \hat{m}_{L}(x_{i})\right), \end{array} $$

where **1**(.) denotes an indicator function; $\hat {m}_{L}$ and $\hat {m}_{U}$ are the lower and upper limits of the pointwise CI, respectively. To asses the fit at the boundary of each simulated function, we additionally computed all these performance indicators also separately for the lower and upper 10% range of the covariate.

To compare the fits graphically, we plotted the mean fitted values of the nonparametric functions and smoothed 95% pointwise coverage probabilities of the true functions. At each observed value of *x*, the mean fitted value was obtained by taking the average over the 1,000 replications. The smoother for coverage probability was obtained using penalized thin plate regression splines while considering logit-transformed coverage probabilities from 1,000 replications as continuous outcome.

#### Results of the simulation study

**Fig. 1:**
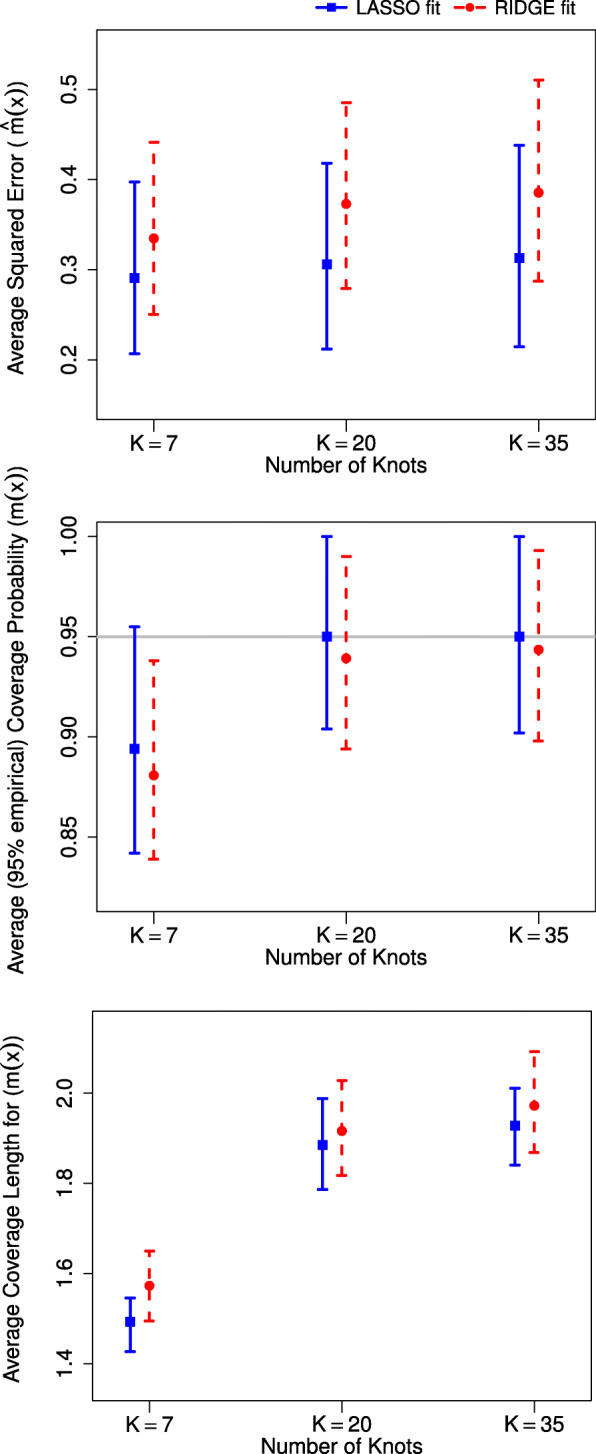
Three performance indicators as a function of number of knots, *K* comparing the performance of using two different penalties (: LASSO and : ridge) for the double hump shape of association. Performance indicators are: Average Squared Error (ASE) of $\hat {m}(x)$, Average Coverage Probability (ACP) and Average Coverage Length (ACL) for *m*(*x*). In all, we present the median and interquartile ranges based on 1,000 replication

Simulation results when two different penalties were used in penalized splines fitting are summarized in Table 2 and, exemplarily for one shape of association (double hump shape), in Fig. 1.

**Table 2:** Simulation results from logistic spline fit by RIDGE and LASSO penalties

	Full Curve			At Boundaries					
				Lower 10*%*			Upper 10*%*		
Penalty	MASE	MACP	MACL	MASE	MACP	MACL	MASE	MACP	MACL
Function : Linear									
**K=7**									
LASSO	0.105	0.96	1.332	0.270	0.96	2.755	0.281	0.96	2.765
RIDGE	0.149	0.96	1.369	0.445	0.95	2.830	0.537	0.95	3.024
**K=20**									
LASSO	0.104	0.96	1.358	0.255	0.96	2.821	0.285	0.97	2.826
RIDGE	0.148	0.96	1.403	0.415	0.95	2.907	0.549	0.96	3.071
**K=35**									
LASSO	0.096	0.96	1.356	0.223	0.96	2.813	0.287	0.97	2.815
RIDGE	0.142	0.96	1.401	0.365	0.96	2.898	0.553	0.96	3.045
Function : Concave									
**K=7**									
LASSO	0.338	0.95	1.791	1.282	0.92	3.461	1.123	0.93	3.467
RIDGE	0.341	0.95	1.786	1.313	0.92	3.465	1.150	0.93	3.471
**K=20**									
LASSO	0.359	0.96	1.953	1.421	0.94	3.886	1.151	0.95	3.787
RIDGE	0.364	0.96	1.951	1.476	0.94	3.979	1.206	0.95	3.855
**K=35**									
LASSO	0.350	0.96	1.958	1.351	0.95	3.873	1.113	0.96	3.829
RIDGE	0.355	0.96	1.953	1.424	0.94	3.965	1.173	0.95	3.846
Function : Double Hump									
**K=7**									
LASSO	0.301	0.90	1.502	0.291	0.92	1.655	0.472	0.92	2.441
RIDGE	0.345	0.89	1.583	0.328	0.92	1.769	0.678	0.91	2.506
**K=20**									
LASSO	0.316	0.95	1.901	0.315	0.94	1.854	0.514	0.95	2.697
RIDGE	0.383	0.94	1.932	0.382	0.93	1.980	0.753	0.93	2.786
**K=35**									
LASSO	0.323	0.95	1.942	0.382	0.95	2.021	0.531	0.95	2.818
RIDGE	0.396	0.94	1.987	0.445	0.94	2.152	0.780	0.93	2.894

We report mean average squared distance (MASE), mean average 95% coverage probability (MACP), and mean average coverage length (MACL) measures for full curve and boundaries for each *K*, penalty and curve

The penalized spline under a mixed model framework using either LASSO or ridge penalty performed well in recapturing the true curves. The mean average squared distances (MASEs) were reasonably small and the mean average coverage probabilities (MACPs) were generally near nominal level in most cases. However, for the linear and complex (double hump) shapes of association, the LASSO penalty overall performed better than the ridge penalty in terms of all performance indicators irrespective of the number of knots considered. For simple (concave) shape of association, both penalties performed quite similarly. At the boundaries (<10*%* and >90*%* of the ranges of *x* values), the LASSO penalty always performed better than ridge penalty in all cases.

As we increased the number of knots (*K*), the overall change in curve fitting performance was not remarkable except for the complex (double hump) shape of association. In general, with larger *K*, the MACP and MACL were larger for both penalties in all considered cases with very few exceptions. The MASE, however, showed different patterns depending on the shape of association. More specifically, for larger *K*, the MASEs were smaller for linear shape of association and larger for double hump shape of association for both penalties. For the concave shape of association, the MASEs fluctuated (for both penalties) as *K* increased.

**Fig. 2:**
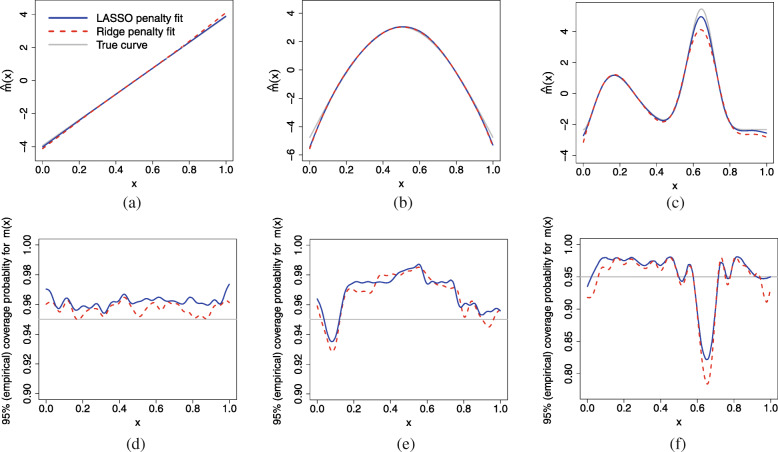
Estimated functions (pointwise mean of fits) vs actual functions in the upper row (**a** linear, **b** concave function, **c** double hump) and smoothed pointwise coverage probabilities of the 95% confidence intervals in the lower row (**d** linear, **e** concave function, **f** double hump) from 1,000 replicated datasets

Figure 2 illustrates the ability of the SPMM using two different penalties to recapture the true functions for *K*=35. The upper panel of Fig. 2 presents the true curves *m*_*j*_(*x*),*j*=1,2,3 and the estimated curves $\hat {m}_{j}(x)$ based on 1,000 replications. The SPMM using either (LASSO or ridge) penalty recovered the true curves reasonably well for all the shapes. However, for the linear association, the SPMM-LASSO fit was relatively close to the true line especially at the borders. For the concave shape, both penalties yielded very similar fits. For the complex (double hump) shape, the reconstructed nonparametric functions had noticeable negative biases when curvature was high. However, the SPMM-LASSO worked well in estimating the high curvature and tail areas as compared to SPMM-RIDGE.

The lower panel of Fig. 2 compares the empirical pointwise coverage probabilities of the 95% confidence intervals of three test functions obtained from SPMMs using two different penalties. For the linear shape of association, the coverage probabilities of the credible intervals from both penalties were slightly above the nominal value of 95%. The SPMM-LASSO coverage probabilities were higher than that of SPMM-RIDGE throughout the range of *x* values. For concave shape, the coverage probabilities from both penalties agreed slightly better with the nominal value (95*%*) throughout the range of *x* except at the boundaries. For the double hump shape, the coverage probabilities of the CIs from both penalties were higher than the nominal value (95*%*), except at boundaries and where biases in the estimated nonparametric functions were noticeable. At *x*-values where the bias was visible, CIs from both penalties yielded low coverage probabilities, but nonetheless SPMM-LASSO had better coverage than SPMM-RIDGE.

Results from the frequentist methods are summarized in Supplementary Tables S2 and S3. Overall, the findings were consistent with that of the Bayesian approach. In most of the simulation scenarios, the Bayesian methods had slightly better performance in recapturing the true curves.

### Sensitivity analysis

We also carried out a number of other simulations using (i) Half-Cauchy(25) priors for the variance components; (ii) truncated quadratic splines ; and (iii) natural cubic splines. For Half-Cauchy (25) priors, the overall results were very close to those reported above. Both the truncated quadratic splines and the natural cubic splines yielded inferior fits as compared to the low-rank thin-plate splines. However, in both cases the LASSO penalty performed better than using the ridge penalty, especially for complex shapes (see Supplementary Table S1).

## Application to COPD data

In this section we used the SPMM to study the association between amount of smoking and risk of chronic obstructive pulmonary disease (COPD). We also investigated the association between the risk of COPD and some occupational variables after adjusting for potential confounders using data from the initial cross-sectional phase of the Canadian cohort of obstructive lung disease (CanCOLD) study [50].

### Methods

#### Data and variables

The data on 6,592 adults aged 40 or above were obtained from the initial (baseline) cross-sectional phase of the prospective longitudinal CanCOLD study. The CanCOLD study is a large, prospective, population-based, multi-site study of COPD. Healthy non smokers, smokers without COPD and subjects with COPD were recruited from nine urban cities across Canada by random telephone digit dialling to identify eligible adults who were then invited to attend a clinic visit to complete questionnaires and to perform prebronchodilator and postbronchodilator spirometry (See [51] for complete details). Data used in this study were collected between August 2005 and May 2009.

We used data from the baseline visit that contained information on subjects’ COPD status (normal, at risk, Global Initiative for Chronic Obstructive Lung Disease (GOLD) stage I, GOLD stage II, GOLD stage III, GOLD stage IV), demographic characteristics, smoking history and occupation. Although there were 6,592 subjects in the study, we excluded 28 participants with the following criteria: (i) reported cigarette pack years less than zero or greater than 150; (ii) participants with implausible BMI values less than 9 or greater than 60; and (iii) reported smoking more than 60 cigarettes per day. We therefore analysed data from 6,564 individuals.

#### Focus on smoking history

While various factors may effectively contribute to the development of COPD, smoking is far and away the primary cause of the disease, according to the World Health Organization (http://www.healthline.com/health/copd/smoking). Thus when looking for other modifiable risk factors for the COPD, it is important to adjust for smoking in the best way possible. An essential measure of smoking intensity is ‘pack years’ calculated as the number of packs smoked per day multiplied by number of smoking years. As the pack years is a continuous variable, its effect on the COPD occurrence may be nonlinear rather than linear. When a nonlinear effect is evident (or apparent), adjusting for a linear effect is likely to lead to residual confounding [52].

We used the SPMM to model the effect of pack years on the risk of COPD (binary outcome, COPD: 0 = no, 1 = yes) nonparametrically. More specifically, we fit the model

16 $$ \begin{aligned} \text{logit}\ \mathbb{P}\left[\text{COPD}_{i} = 1\mid \text{pack years}_{i}\right] = \beta_{0} + m_{1}(\text{pack years}_{i}), \end{aligned}  $$

where *β*_0_ is the intercept of the model, *m*_1_ is some smooth function of pack years and $i = 1, \dots, 6564$.

We then evaluated the effect of occupational exposures (such as asbestos, chemical manufacturing, welding, hard rock mining, coal mining) on COPD, one by one, with and without adjusting for the effects of potential confounders: pack years, age, sex, and BMI. Note that due to the multiple response allowed for the occupational exposure, different occupational categories considered in this study were not mutually exclusive. For example, an individual working in a steel mill/factory was also reported in the occupational exposure group of welding, and chemical/plastic manufacturing. As such, we could not include the occupation as a single categorical variable in the model. For estimating the adjusted effect we fit the model

17 $$ {}\begin{aligned} \text{logit}\ \mathbb{P}\left[\text{COPD}_{i}\right. &=\left. 1\mid \text{covariates}_{i}\right]\,=\, x^{T}_{i}\beta \!+ m_{1}(\text{pack years}_{i}) \\ &\quad+ m_{2}(\text{age}_{i}) + m_{3}(\text{BMI}_{i}),\\ \end{aligned}  $$

where *m*_*j*_,*j*=1,2,3 are smooth functions, *x*_*i*_ are fixed effect covariates that include an intercept, a binary (yes/no) occupation variable and sex, and *β* are fixed effect parameters.

Following simulation results, which suggested that the Bayesian SPMM using LASSO penalty had relatively better curve fitting performance than other frequentist and Bayesian methods, we only adopted the Bayesian SPMM imposing LASSO type penalty for curve fitting. Each of the smooth functions in (16) and (17) was estimated by using penalized low-rank thin-plate regression splines with a large number of knots *K*=20, where knot positions were specified as in (15). Considering LASSO penalty, we imposed the centering constraint on each smoother such that the sum of the elements of each smoother *m*_*j*_(.) is zero (see, [14] for details). Representing each smoother as a mixed model component, we estimate the model parameters using a Bayesian approach via MCMC sampling. Noninformative prior distributions were used for all fixed effects and variance components (*N*(0,10^6^) and Uniform(0,100), respectively). To estimate each model, we ran 2 chains and the estimates were medians from 55,000 iterations after discarding the initial 5,000 iterations of burn-in. Both chains were thinned by keeping every 50th iteration. A 95% posterior credible interval for each parameter of interests was obtained as 2.5th and 97.5th percentiles of the posterior sample. We evaluated convergence of the chains by visually examining the trace plot, density plot, sample autocorrelation function for each parameter, and also following Gelman and Rubin [53] to quantify the between-chain and the within-chain variability of a quantity of interest.

#### Results of the data analysis

Table 3 presents the demographic characteristics, smoking behavior and occupational exposure types in the entire cohort, stratified by COPD status. Approximately 21% participants were diagnosed with COPD. Compared to non-COPD participants, COPD participants were older, included more men, more smokers, and more pack years of smoking, had a higher proportion of smokers who had quit smoking, and had a slightly higher proportion of occupational exposure to hard rock, coal, asbestos, chemical, steel, welding and saw-milling.

**Table 3:** Characteristics of the participants/study population, by COPD status

Characteristic	Summary Measure (total n = 6,564)			
	COPD		NO COPD	
	n = 1367	(20.8%)	n = 5197	(79.2%)
Age	65.2	(11.2)	57.0	(10.8)
Male	755	(55.2%)	2286	(44.0%)
BMI	27.3	(5.3)	27.9	(5.7)
Ever smoker (cigarette)	943	(69.0%)	2623	(50.5%)
Ever smoker (pipe or cigarette)				
Never smoker	406	(29.7%)	2543	(48.9%)
Ex smoker	645	(47.2%)	2030	(39.1%)
Current smoker	316	(23.1%)	624	(12.0%)
Pack Years	22.9	(24.6)	10.5	(17.0%)
Average cigarette per day	13.2	(12.6)	8.3	(11.4)
Duration of smoking (year)	23.0	(19.7)	11.8	(14.9)
Smoking cessation	943	(69.0%)	2623	(50.5%)
Occupation				
Hard rock mining	34	(2.5%)	80	(1.5%)
Coal mining	11	(0.8%)	12	(0.2%)
Working with asbestos	59	(4.3%)	157	(3.0%)
Chemical/plastics manufacturing	80	(5.9%)	231	(4.4%)
Foundry/steel milling	39	(2.9%)	106	(2.0%)
Welding	68	(5.0%)	172	(3.3%)
Saw-milling	39	(2.9%)	103	(2.0%)

Mean (SD) is reported for quantitative variables, while count (*%*) is reported for categorical variables

For all models fit via MCMC simulation, we observed good mixing properties of the chains with fat hairy caterpillars like trace plots, similar density plots, and few significant autocorrelations. The Gelman-Rubin $\sqrt {(\hat {R})}$ values [53] for the estimates were all less than 1.03 indicating good convergence of the chains.

**Fig. 3:**
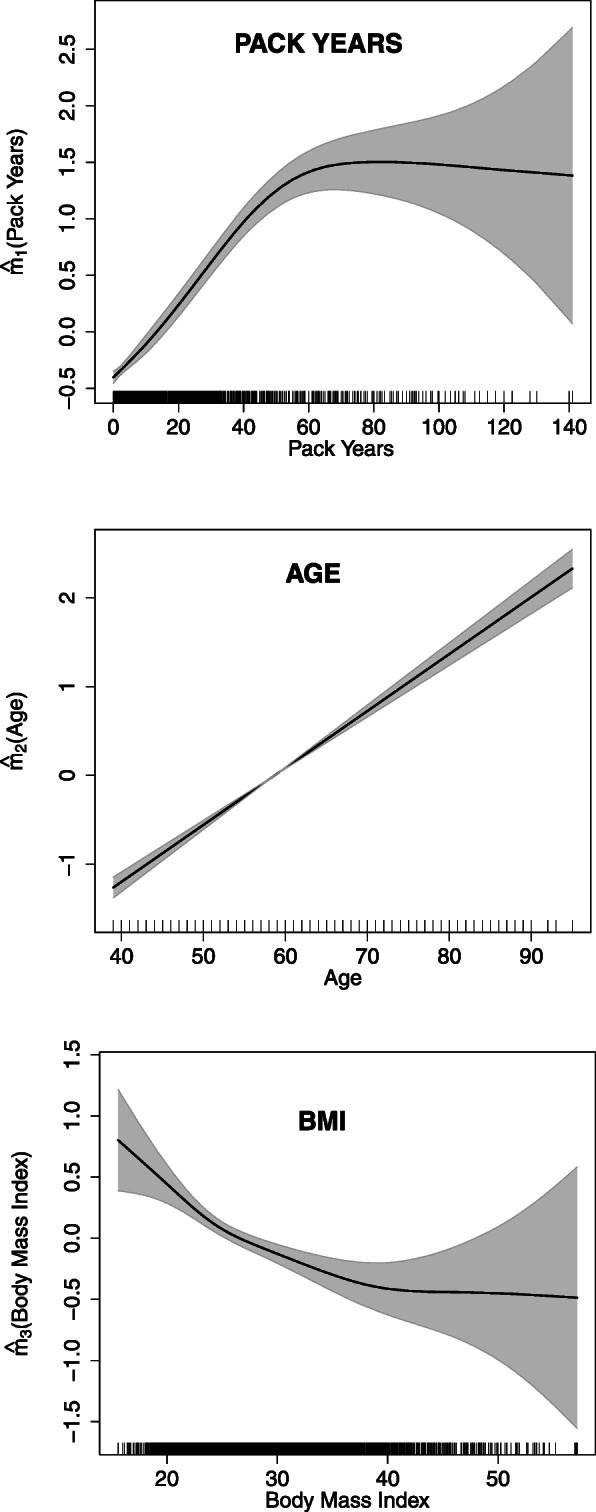
LASSO type penalized splines estimates of *m*_1_(pack years), *m*_2_(age) and *m*_3_(BMI) for the logit of the prevalence of COPD. The shaded regions are the pointwise 95% credible sets obtained from the fully Bayesian fit

The first panel of Fig. 3 shows the estimated nonparametric functions of pack years obtained from fitting model (16) using a LASSO penalty. It is clearly apparent that the association between pack years and COPD was nonlinear. The risk of COPD increased sharply until about 60 pack years and then flattened out.

Results from all models fits to evaluate the association between occupational variables and COPD are summarized in Table 4. All of the considered occupational variables including hard rock mining, coal mining, working with asbestos, chemical/plastics manufacturing, foundry/steel milling, welding, saw-milling were found to have a statistically significant impact on the risk of the prevalence of COPD when considering unadjusted models. However, none of these variables significantly affected COPD when the models were properly adjusted for the effects of potential confounders.

**Table 4:** Results from SPMMs fit using LASSO penalty for assessing the effect of occupational exposures on the occurrence of COPD

	Odds Ratio (95% CI) ^*a*^	
	Unadjusted	Adjusted
Age	-	*
Pack Years	-	*
BMI	-	*
Sex (Men)	-	**
Occupation		
Hard rock mining	1.6 (1.1, 2.4)	1.1 (0.7, 1.8)
Coal mining	3.5 (1.5, 8.0)	1.7(0.7, 4.3)
Working with asbestos	1.4 (1.1, 2.0)	0.8 (0.5, 1.1)
Chemical/plastics manufacturing	1.3 (1.1, 1.7)	1.1 (0.8, 1.5)
Foundry/steel milling	1.4 (1.0, 2.0)	1.0 (0.7, 1.5)
Welding	1.5 (1.1, 2.0)	1.2 (0.9, 1.6)
Saw-milling	1.5 (1.0, 2.1)	1.2 (0.8, 1.8)

^a^Adjusted ORs were obtained from the SPMM as shown in (17) adjusting for pack years, age, sex and BMI

^*^ Covariates were modeled using penalized splines. A summary of odds ratio and CI were not provided by the method; see Fig. 3 for smooth curves when occupation was hard rock mining

^**^ OR = 1.4 (1.3, 1.6) when occupation was hard rock mining

The estimated shape of the pack years-COPD association from the adjusted model (17) (where occupational variable was hard rock mining) was the same as shown in the first panel of Fig. 3. Also, the estimated nonparametric functions of age and BMI from adjusted model are shown in the second and third panels of Fig. 3, respectively. It is evident that the age of the participants was linearly associated with COPD prevalence whereas the BMI-COPD association was slightly nonlinear. Low BMI was associated with an increased risk, and a lowered risk with increasing BMI plateaued off at BMI 40.

## Discussion

Under the semiparametric mixed model (SPMM), we introduced the LASSO type absolute penalty to investigate if the performance of curve fitting can be improved over that using a typical ridge penalty.

We adopted a fully Bayesian approach to estimate SPMMs for binary outcomes. Via simulations, we assembled evidence suggesting that using a LASSO penalty is an eligible competitor to the typical ridge regression type penalty. We evaluated the relative performance of the penalties in three different scenarios: linear, simple (concave function) and complex (double hump) shapes of association.

Test results suggested that the LASSO penalty performed better than the ridge penalty in recapturing the linear and complex functional forms between continuous predictor and binary outcome. For simpler nonlinear association both penalties performed similarly. These results may be due to the fact that, for linear association, there are a large number of very small regression coefficients at knots to be penalized and the LASSO penalty does a better job in shrinking them towards zero. Tibshirani [32] showed that the LASSO defines a continuous shrinking operation that can produce coefficients that are exactly zero. Relatively more shrinkage of all small knot coefficients towards zero leads to a straight line fit rather than curvature. For the complex shape, there are a small to moderate number of large or moderate-sized regression coefficients at knots. In such cases, the LASSO penalty puts more weight on large coefficients and, hence, may perform better to estimate the curvature areas as compared to the ridge penalty, which penalizes all regression coefficients almost uniformly. Finally, for the simple nonlinear shape (concave function) of association, there are a large number of very small or moderate-sized coefficients to be shrunk and in such situations both penalties perform similarly.

Note that in a linear regression setting, LASSO is a worthy competitor to subset selection and ridge regression, outperforming other methods when there are a small to moderate number of moderate-sized effects [32]. Similarly, the LASSO penalized SPMMs for curve fitting does not significantly outperform its closest contender the ridge penalty in all cases but performs better in scenarios where the curvature is high or the association is linear, although the LASSO penalty yielded better fit around boundaries in all considered cases.

Overall, we found that the Bayesian estimates of the nonparametric functions were not very sensitive to the choice of prior distributions for the variance components. Results from using uniform priors for variance components were very similar to those obtained using half-Cauchy priors. Low-rank thin-plate splines were found to perform better than natural cubic splines or truncated quadratic splines, confirming the results in Crainiceanu et al. [15]: a good choice of basis function has important consequences for the mixing properties of the MCMC chains in Bayesian analysis.

We also found that the Bayesian methods had marginally better performance than the popular frequentist methods for curve fitting in most cases. However, because of the differences in estimation techniques, representation of smooth functions, choice of splines and basis functions, selection of smoothing parameter, the observed differences in frequentist and Bayesian methods may be due to any number of reasons, not necessarily the type of penalty imposed.

We applied the proposed Bayesian method to identify the association between some occupational variables and chronic obstructive pulmonary disease (COPD) after investigating the shape of the smoking-COPD association using data from the Canadian cohort of obstructive lung disease (CanCOLD) study. We found that the smoking-COPD association was nonlinear. To identify potential occupational risk factors for COPD, while minimizing the risk of residual confounding, it is crucial to adjust for smoking appropriately and that seemed to be using a smooth function given that the shape is not linear. After adjusting for smoking pack years, age, sex and BMI, we found all occupational variables had statistically insignificant effects on COPD.

In this paper, we included both simulated and real datasets. However, the range of simulation scenarios investigated was not exhaustive. For simplicity, and to avoid intensive computational efforts, we smoothed only one covariate in the simulation. The extension to smooth multiple covariates in a SPMM is straightforward and we demonstrated this on an example from CanCOLD-COPD data.

## Conclusion

The promising results from this study suggest that the LASSO penalty might better capture complex dose-response association. However, the Bayesian estimation of SPMM using LASSO penalty is relatively complex and time consuming; it might take relatively three-fold more time compared to ridge penalty.

## Supplementary Information

**Additional file 1** Supplementary Material: This file contains three tables that summarize additional simulation results and R code.

## Data Availability

In this paper we use secondary data from the initial cross-sectional phase of the Canadian cohort of obstructive lung disease (CanCOLD) study. The data are not available online and required administrative permission. We were granted permission for using this data by Dr. Jean Bourbeau.

## Abbreviations

GLMM: Generalized linear mixed model
SPMM: Semiparametric mixed model
LASSO: Least absolute shrinkage and selection operator
MCMC: Markov Chain Monte Carlo
COPD: Chronic obstructive pulmonary disease
LRTP: Low-rank thin-plate spline
DE: Double-exponential
PQL: Penalized quasi-likelihood
IG: Inverse-gamma
JAGS: Just another Gibbs sampler
MASE: Mean average squared error
MACP: Mean average coverage probability
MACL: Mean average confidence interval length
BMI: Body mass index
CanCOLD: Canadian cohort of obstructive lung disease
