# Supplementary material for: LASSO type penalized spline regression for binary data
Source: BMC Med Res Methodol. 2021 Apr 24;21:83. doi: 10.1186/s12874-021-01234-9 (PMC8070328; doi:10.1186/s12874-021-01234-9)
Supplement: Supplementary file 1 — Additional file 1 Supplementary Material: This file contains three tables that summarize additional simulation results and R code. [file 12874_2021_1234_MOESM1_ESM.pdf]

LASSO type Penalized Spline Regression for Binary Data

Muhammad Abu Shadeque Mullah[1], James A Hanley[1] and Andrea Benedetti*[1,2]

[1]Department of Epidemiology, Biostatistics and Occupational Health, McGill University

[2]Department of Medicine, McGill University, and Respiratory Epidemiology & Clinical Research Unit, Montreal Chest Institute, McGill University Health Centre

*Corresponding Author: Associate Professor, Department of Medicine and of Epidemiology, Biostatistics and Occupational Health, McGill University, E-mail: andrea.benedetti@mcgill.ca

1 Supplementary Materials

Table S1: Simulation results from sensitivity analysis: logistic splines fit by RIDGE and LASSO penalties to recapture the double hump shape of association. We report mean average squared distance (MASE), mean average 95% coverage probability (MACP), and mean average coverage length (MACL) measures for full curve and at boundaries for each K, prior, and basis function.

Splines: Low-rank thin-plate; Priors: Half-Cauchy (25)									
	Full Curve			At Boundaries					
				Lower 10%			Upper 10%		
Penalty	MASE	MACP	MACL	MASE	MACP	MACL	MASE	MACP	MACL
**K=7**									
LASSO	0.304	0.89	1.436	0.319	0.91	1.594	0.476	0.91	2.374
RIDGE	0.349	0.88	1.513	0.359	0.91	1.704	0.682	0.90	2.436
**K=20**									
LASSO	0.320	0.94	1.823	0.328	0.93	1.784	0.518	0.94	2.623
RIDGE	0.388	0.93	1.852	0.386	0.92	1.905	0.758	0.92	2.709
**K=35**									
LASSO	0.326	0.94	1.876	0.387	0.94	1.942	0.537	0.95	2.745
RIDGE	0.400	0.93	1.919	0.449	0.93	2.067	0.786	0.93	2.818
Splines: Truncated quadratic; Priors: Uniform (0, 100)									
**K=7**									
LASSO	0.733	0.76	1.307	0.312	0.92	1.760	0.480	0.90	2.388
RIDGE	0.840	0.75	1.378	0.352	0.92	1.824	0.690	0.89	2.452
**K=20**									
LASSO	0.511	0.83	1.518	0.364	0.92	1.783	0.534	0.93	2.525
RIDGE	0.619	0.82	1.543	0.442	0.91	1.904	0.783	0.92	2.608
**K=35**									
LASSO	0.403	0.85	1.563	0.383	0.94	1.867	0.544	0.94	2.643
RIDGE	0.494	0.84	1.599	0.446	0.93	1.987	0.799	0.93	2.714
Splines: Natural cubic; Priors: Uniform (0, 100)									
**K=7**									
LASSO	1.443	0.40	1.071	0.924	0.30	1.071	0.650	0.90	2.367
RIDGE	2.617	0.24	1.069	1.193	0.26	1.062	4.306	0.56	3.110
**K=20**									
LASSO	1.379	0.41	1.130	0.741	0.42	1.127	0.766	0.90	2.576
RIDGE	2.358	0.36	1.090	0.747	0.39	1.122	3.668	0.75	2.914
**K=35**									
LASSO	1.287	0.47	1.184	0.505	0.55	1.182	0.881	0.91	2.844
RIDGE	1.653	0.44	1.087	0.528	0.54	1.175	0.868	0.79	2.876

Table S2: Simulation results from logistic spline fit by different methods: Bayesian Semiparametric Mixed Model (SPMM) with LASSO Penalty; Bayesian SPMM with RIDGE Penalty; Generalized Additive Model (GAM) as implemented in `R` package `gam`; and GAM as implemented in `R` package `mgcv`. We report mean average squared distance (MASE), mean average 95% coverage probability (MACP), and mean average coverage length (MACL) measures for full curve and also at boundaries.

	Full Curve			At Boundaries					
				Lower 10%			Upper 10%		
Method	MASE	MACP	MACL	MASE	MACP	MACL	MASE	MACP	MACL
**Function : Linear**									
**K=7**									
SPMM with LASSO Penalty	0.105	0.96	1.332	0.270	0.96	2.755	0.281	0.96	2.765
SPMM with RIDGE Penalty	0.149	0.96	1.369	0.445	0.95	2.830	0.537	0.95	3.024
GAM in gam Package (df = 7)	0.329	0.98	1.905	1.051	0.98	3.836	1.259	0.98	3.883
GAM in mgcv Package	0.234	0.95	0.957	0.592	0.96	1.592	1.220	0.96	1.651
**K=20**									
SPMM with LASSO Penalty	0.104	0.96	1.358	0.255	0.96	2.821	0.285	0.97	2.826
SPMM with RIDGE Penalty	0.148	0.96	1.403	0.415	0.95	2.907	0.549	0.96	3.071
GAM in gam Package (df = 20)	2.691	0.98	4.484	10.478	0.98	11.932	12.192	0.98	12.197
GAM in mgcv Package	0.420	0.96	1.094	0.922	0.96	1.919	2.389	0.96	2.207
**K=35**									
SPMM with LASSO Penalty	0.096	0.96	1.356	0.223	0.96	2.813	0.287	0.97	2.815
SPMM with RIDGE Penalty	0.142	0.96	1.401	0.365	0.96	2.898	0.553	0.96	3.045
GAM in gam Package (df = 35)	13.347	0.98	11.258	53.871	0.98	38.529	63.651	0.98	39.884
GAM in mgcv Package	0.223	0.96	1.133	0.721	0.96	2.066	0.861	0.96	2.127
**Function : Concave**									
**K=7**									
SPMM with LASSO Penalty	0.338	0.95	1.791	1.282	0.92	3.461	1.123	0.93	3.467
SPMM with RIDGE Penalty	0.341	0.95	1.786	1.313	0.92	3.465	1.150	0.93	3.471
GAM in gam Package (df = 7)	0.256	0.98	1.985	0.782	0.97	3.645	0.676	0.97	3.531
GAM in mgcv Package	0.207	0.95	1.444	0.764	0.93	2.576	0.581	0.93	2.523
**K=20**									
SPMM with LASSO Penalty	0.359	0.96	1.953	1.421	0.94	3.886	1.151	0.95	3.787
SPMM with RIDGE Penalty	0.364	0.96	1.951	1.476	0.94	3.979	1.206	0.95	3.855
GAM in gam Package (df = 20)	1.885	0.98	4.287	7.890	0.98	10.563	6.100	0.98	9.032
GAM in mgcv Package	0.740	0.96	1.553	2.016	0.93	2.809	4.289	0.94	2.783
**K=35**									
SPMM with LASSO Penalty	0.350	0.96	1.958	1.351	0.95	3.873	1.113	0.96	3.829
SPMM with RIDGE Penalty	0.355	0.96	1.953	1.424	0.94	3.965	1.173	0.95	3.846
GAM in gam Package (df = 35)	9.114	0.98	9.756	41.300	0.98	32.293	31.533	0.98	25.866
GAM in mgcv Package	0.194	0.96	1.522	0.6332	0.94	2.679	0.522	0.94	2.628
**Function : Double Hump**									
**K=7**									
SPMM with LASSO Penalty	0.301	0.90	1.502	0.291	0.92	1.655	0.472	0.92	2.441
SPMM with RIDGE Penalty	0.345	0.89	1.583	0.328	0.92	1.769	0.678	0.91	2.506
GAM in gam Package (df = 7)	0.928	0.74	1.310	0.198	0.90	1.515	0.522	0.98	2.304
GAM in mgcv Package	0.733	0.68	1.283	0.206	0.88	1.482	0.567	0.79	2.005
**K=20**									
SPMM with LASSO Penalty	0.316	0.95	1.901	0.315	0.94	1.854	0.514	0.95	2.697
SPMM with RIDGE Penalty	0.383	0.94	1.932	0.382	0.93	1.980	0.753	0.93	2.786
GAM in gam Package (df = 20)	0.416	0.98	2.703	0.629	0.98	2.732	0.827	0.98	3.347
GAM in mgcv	0.555	0.92	1.896	0.349	0.96	2.040	0.752	0.97	2.684
**K=35**									
SPMM with LASSO Penalty	0.323	0.95	1.942	0.382	0.95	2.021	0.531	0.95	2.818
SPMM with RIDGE Penalty	0.396	0.94	1.987	0.445	0.94	2.152	0.780	0.93	2.894
GAM in gam Package (df = 35)	1.509	0.98	4.548	1.720	0.98	4.085	2.387	0.98	5.160
GAM in mgcv Package	0.534	0.92	1.950	0.363	0.96	2.095	0.532	0.98	2.675

Table S3: Simulation results from logistic spline fit by two frequentist methods as implemented in `R` software: (i) Generalized Additive Model (GAM) in `gam` package (ii) GAM in `mgcv` package using the default in both packages. We report mean average squared distance (MASE), mean average 95% coverage probability (MACP), and mean average coverage length (MACL) measures for full curve and also at boundaries.

Function : Linear									
	Full Curve			At Boundaries					
				Lower 10%			Upper 10%		
Method	MASE	MACP	MACL	MASE	MACP	MACL	MASE	MACP	MACL
GAM in `gam` Package	0.151	0.98	1.441	0.435	0.98	2.797	0.496	0.98	2.861
GAM in `mgcv` Package	0.121	0.95	0.983	0.368	0.96	1.658	0.386	0.96	1.709
Function : Concave									
GAM in `gam` Package	0.117	0.97	1.422	0.310	0.94	2.414	0.288	0.95	2.377
GAM in `mgcv` Package	0.311	0.95	1.497	1.431	0.93	1.592	0.894	0.93	2.626
Function : Double Hump									
GAM in `gam` Package	2.345	0.28	0.920	0.906	0.29	1.089	0.563	0.86	1.861
GAM in `mgcv` Package	0.313	0.92	1.660	0.310	0.94	1.831	0.568	0.96	2.481

R Code for LASSO type Penalized Splines

```
K <- 20 # or 7, 35
library(MCMCglmm)
Z.matrix <- spl(x,  k = K, knots = NULL, type = "LRTP") # thin plate splines
Z <- as.matrix(Z.matrix)
X <- cbind(rep(1, dim(data)[1]), data$x)
Ncovariate <- dim(X)[2]
Nknots <- dim(Z)[2]
y <- data$y

# MODEL FOR SPMM-LASSO
cat(
"
model{
for(i in 1:N)
{
y[i] ~ dbern(p[i])
logit(p[i]) <- inprod(beta[], X[i, ]) + inprod(b[], Z[i, ])
}
for(j in 1:Nknots)
{
b[j] ~ ddexp(0, taub)
}
for(j in 1:Ncovariate)
{
beta[j] ~ dnorm(0, 0.000001)
}
taub    <-  1/(sigmab)
sigmab  ~  dunif(0.000001, 100)
logit.p <- log(p/(1-p))
}
"
, fill = TRUE, file = "jagsmodel.txt")
```
